# Understanding the struggles of first-generation medical students and interns: a cross-sectional study in Saudi Arabia

**DOI:** 10.25122/jml-2024-0310

**Published:** 2024-10

**Authors:** Zainab Alsuni, Asmah Alhubaishi, Ghaliah Othman, Fatemah Alghanem, Abeer Zakariyah, Jameel Bardesi, Hesham Rizk

**Affiliations:** 1 College of Medicine, University of Jeddah, Jeddah, Saudi Arabia; 2 Department of Medical Genetics, College of Medicine, University of Jeddah, Jeddah, Saudi Arabia; 3 Department of Radiology, College of Medicine, University of Jeddah, Jeddah, Saudi Arabia; 4 Department of Surgery, College of Medicine, University of Jeddah, Jeddah, Saudi Arabia

**Keywords:** First-generation medical students, stress, burnout, challenges, Saudi Arabia

## Abstract

This study explored the experiences of first-generation and non-first-generation medical students in Saudi Arabia regarding their education, career aspirations, attitudes toward medical school, and perceived stress. We aimed to provide insights into the struggles faced by first-generation medical students. This cross-sectional study was conducted with 485 participants, 77.9% of whom were first-generation students. Data were collected using a questionnaire adapted from the Association of American Medical Colleges (AAMC) and analyzed using chi-square and Mann–Whitney tests. First-generation students were less decisive about future specialties, showed greater interest in taking the United States Medical Licensing Examination and the Professional and Linguistic Assessments Board exams, and expressed less interest in teaching roles than their non-first-generation counterparts. No significant differences were found in attitude, career considerations, quality of life, or daily activities. This study aligns with global calls for robust support programs, mentorship initiatives, and systemic interventions to enhance diversity and inclusivity in medical education. The research highlighted the importance of recognizing the diverse career aspirations and challenges first-generation medical students face. Tailored support programs are essential for fostering inclusivity in medical education, addressing unique needs, and enhancing students’ overall well-being. Future research should continue to explore the factors influencing the experiences of first-generation and non-first-generation medical students to contribute to ongoing efforts to improve medical education.

## INTRODUCTION

First-generation (FG) medical students, defined as individuals who are the first in their families to attend medical school, face unique challenges compared to their non-first-generation (NFG) peers [1]. Studies have shown that FG medical students have a lower quality of life, higher levels of exhaustion and stress, significant financial hardships, and limited social support [1,2]. Other studies mentioned that they also needed help in several areas as they needed more resources than their peers, inadequate support from their institutions and faculty members, and felt isolated within the medical school [3].

Emotional fatigue and a reduced likelihood of practicing self-care contribute to a lower environmental quality of life among FG students compared to their NFG counterparts [2]. For example, a previous study in the United States found that FG medical students experienced poorer sleep quality and higher levels of somnolence because of insufficient sleep. As a result, initiatives in the United States have emerged to address the concerns expressed by FG medical students [1,4]. Furthermore, FG medical students had a greater need for role models and institutional recognition of their family and personal needs [5].

A growing body of research also highlights the social and professional disparities FG students face. FG students often report limited access to research opportunities, mentors with similar backgrounds, and opportunities to shadow physicians. They are more likely to enter workforce shortages and serve underprivileged communities [6]. Furthermore, these students frequently experience feelings of exclusion, not fitting in, and being perceived as different, inferior, misunderstood, or marginalized. These feelings were often unintentionally triggered by the comments and behaviors of their peers or instructors, who were NFG students. The participants identified three main aspects of their identity that contributed to their experience of otherness: FG status, socioeconomic status, and race and ethnicity, often used interchangeably by the students [6,7].

A study conducted in the Western Region of Saudi Arabia focused on depression among FG and NFG medical students [8]. However, broader research across all regions of Saudi Arabia is needed to understand depression among medical students better. A German study examined factors influencing medical students’ career choices, such as background and educational experiences, and found that students often gravitated towards specialties offering a better work-life balance. While the study did not specifically target FG students, it reflected challenges that may be relevant to them, such as the need for job satisfaction and balance in career planning [9]. Similarly, a UK study of 42 medical schools showed significant variation in students’ career intentions, with many expressing dissatisfaction with the National Health Service (NHS) and a desire to leave the profession shortly after graduation. Although this study did not focus on FG students, it highlighted potential issues such as a lack of professional networks, which FG students may have experienced more acutely, influencing their career satisfaction and retention within the medical field [10].

To date, insufficient research has been conducted in Saudi Arabia to explore the connection between being an FG medical student and encountering specific challenges and hurdles. This study seeks to address this gap by examining the correlation between FG status and the obstacles medical students face in the Saudi context. By identifying these challenges, this research underscores the importance of establishing robust support systems to empower FG medical students and promote their success in medical education.

## MATERIAL AND METHODS

### Study design and sample size calculation

This cross-sectional study was conducted from September to November 2023 and included undergraduate medical students and interns from all universities in Saudi Arabia. The sample size was calculated based on a population size of 7,992, as reported in the Ministry of Health's Statistical Yearbook in 2021 [11]. Using the Raosoft online sample size calculator, a minimum of 367 participants was determined to provide a 5% margin of error with a 95% confidence level, ensuring a representative sample. All medical students and interns who attended medical school, whether private or governmental, in Saudi Arabia, were included in the study. However, those who pursued medical education outside the borders of Saudi Arabia were excluded.

### Questionnaire

The questionnaire used in this study was adapted from the Association of American Medical Colleges (AAMC) Medical School Year Two Questionnaire (Y2Q) [12], designed to capture medical students' experiences during their second year of education. The questionnaire covered multiple domains, including demographic information, academic records, preferred general specialties, identification as FG medical students, aspirations for future postgraduate studies, career preferences, involvement in full-time patient care and research, willingness to reconsider their medical school choice, attitudes towards and satisfaction with medical school, and post-medical school career path considerations. Additionally, assessments were conducted to ascertain the daily hours allocated to various activities. Participants' quality of life (QOL) and perceived stress levels were assessed using the Perceived Stress Scale 4 (PSS-4). PSS-4 scores range from 0 to 16, with higher scores indicating increased stress levels. Scores of six or higher were categorized as high stress based on population norms. The questionnaire was distributed to medical students and interns in Saudi Arabia through various social media platforms, including WhatsApp, Twitter, and Telegram. Utilizing shared groups ensured access to the FG and NFG student populations, mitigating potential selection bias. The questionnaire was divided into two parts to minimize selection bias: one tailored for medical students and the other for interns. This approach ensured that the questions were relevant and appropriate for each group, enhancing the accuracy and validity of the collected data. The internal consistency of the questionnaire was examined, and a Cronbach's alpha value of 0.82 was determined.

### Data analysis

Statistical analyses were conducted using SPSS version 26. The chi-square test (χ^2^) was used to analyze qualitative data presented as numbers and percentages to investigate variable associations. The Mann–Whitney test was used for quantitative non-parametric variables expressed as the mean and standard deviation (mean ± SD). Correlation analysis was performed using Spearman's test, with statistical significance set at a *P* value of less than 0.05.

## RESULTS

### Sociodemographic characteristics of the participants

Of the 485 undergraduates and interns, 107 (22.1%) were identified as NFG medical students, and the remaining 378 (77.9%) were categorized as FG medical students. Among the participants, 452 (93.2%) were undergraduates, and 33 (6.8%) were interns. The mean age was 22.33 ± 3.7 years. Most participants (97.3%) held Saudi nationality, 96.1% were single, and 76.7% had no dependents (including a spouse). The majority (83.1%) had a monthly income of less than 5,000 SR. Of these, 40.6% had a GPA of 4.5-5 out of 5, and 17.7% had a GPA of 3.5-4 out of 4 (Table 1).

**Table 1:** Distribution of undergraduates and interns according to their demographic and academic data (*n* = 485)

Variable	Frequency (%)
**Academic status**	
Intern Undergraduate	33 (6.8) 452 (93.2)
**Age**	22.33 ± 3.7
**Gender**	
Female Male	289 (59.6) 196 (40.4)
**Nationality**	
Non-Saudi Saudi	13 (2.7) 472 (97.3)
**What is your current marital status?**	
Divorced Married Single	6 (1.2) 13 (2.7) 466 (96.1)
**How many dependents do you have (including a spouse)?**	
None 1 2 3 ≥4	372 (76.7) 33 (6.8) 28 (5.8) 18 (3.7) 34 (7)
**Your monthly estimated income in Saudi Riyals:**	
<5000 SR 5000-<10000 10000-15000 >15000	403 (83.1) 76 (15.7) 2 (0.4) 4 (0.8)

### Participants’ future specialty preferences

**Figure 1:**
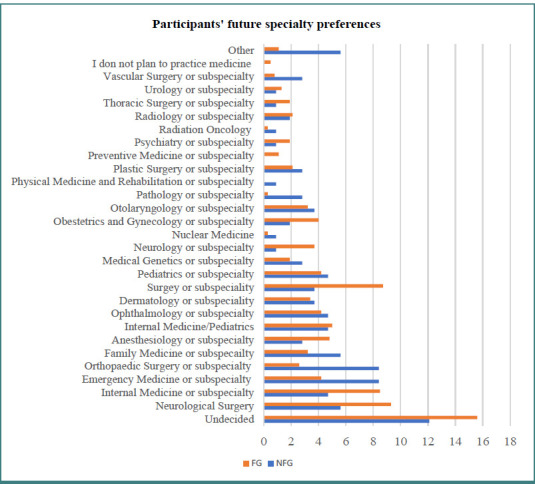
**Comparison between FG and NFG undergraduates and interns according to the general specialty**. The figure compares the distribution of general specialty preferences between FG and NFG medical students, including both undergraduates and interns. FG; first generation, NFG; non first generation.

A comparison of future specialty preferences between FG and NFG undergraduates and interns revealed that FG participants were more likely to be undecided about their future general specialty than their NFG peers, as shown in Figure 1.

### Postgraduate studies and career preferences

FG participants expressed significantly greater interest in taking the United States Medical Licensing Examination (USMLE) or the Professional and Linguistic Assessments Board (PLAB) exams compared to NFG participants (*P* < 0.05, Table 2). Furthermore, a significantly lower percentage of FG participants expressed willingness to participate in teaching roles during their careers (*P* < 0.05, Table 3).

**Table 2:** Comparison of FG and NFG medical students regarding future postgraduate board exams

Variable		Are you a first-generation medical student?		χ^2^	*P* value
		No *n* = 107	Yes *n* = 378		
**Are you planning to take any board exams other than SMLE (e.g., USMLE)?**					
I don’t know	226 (46.6)	48 (44.9)	178 (47.1)	0.17	0.915
No	98 (20.2)	22 (20.6)	76 (20.1)		
Yes	161 (33.2)	37 (34.6)	124 (32.8)		
**If you have taken any board exams other than the Saudi Medical Licensing Examination (SMLE), please specify which one. (Total responses: 161)**					
Canadian board exam	3 (1.8)	0 (0.0)	3 (0.8)	22.19	0.014
CaRMS	1 (0.6)	1 (0.9)	0 (0.0)		
GMAT	1 (0.6)	0 (0.0)	1 (0.3)		
MCCQE	7 (4.3)	0 (0.0)	7 (1.9)		
PLAB	2 (1.2)	7 (6.5)	39 (10.3)		
USMLE	83 (58.1)	0 (0.0)	2 (0.5)		
USMLE, MCCQ	6 (3.7)	19 (17.8)	64 (16.9)		
USMLE, PLAB	1 (0.6)	5 (4.7)	1 (0.3)		
USMLE, UCAT	1 (0.6)	0 (0.0)	1 (0.3)		
NA	46 (28.5)	0 (0.0)	1 (0.3)		

The table compares the plans of FG and NFG medical students to take postgraduate board exams other than SMLE (e.g., USMLE). Percentages represent the proportion of students in each category. Chi-square (χ^2^) values are shown for significant group differences, with *P* values indicating statistical significance (*P* < 0.05).

SMLE, Saudi Medical Licensing Examination; CaRMS, Canadian Resident Matching Service; GMAT, Graduate Management Admission Test; MCCQE, Medical Council of Canada Qualifying Examination; PLAB, Professional and Linguistic Assessments Board (exam for practicing medicine in the UK); USMLE, United States Medical Licensing Examination; UCAT, University Clinical Aptitude Test; NA, Not Applicable.

**Table 3:** Comparison of FG and NFG medical students’ future career preferences

Variable		Are you a FG medical student?		χ^2^	*P* value
		No (*n* = 107)	Yes (*n* = 378)		
**Which of the following activities do you intend to pursue during your medical career?**					
**Patient care**	410 (84.5)	86 (80.4)	324 (85.7)	1.81	0.177
**Research**	367 (75.7)	77 (72)	290 (76.7)	1.02	0.311
**Public health**	204 (42.1)	46 (43)	158 (41.8)	0.04	0.826
**Teaching**	141 (29.1)	21 (19.6)	120 (31.7)	5.94	0.015
**Medical school faculty**	171 (35.3)	34 (31.8)	137 (36.2)	0.72	0.393
**Administration (e.g., Department Chair, Dean)**	61 (12.6)	10 (9.3)	51 (13.5)	1.3	0.254
**Military service**	28 (5.8)	8 (7.5)	20 (5.3)	0.73	0.392

The table compares future career preferences between FG and NFG medical students. Percentages show the proportion of students choosing each activity. Chi-square (χ^2^) values test group differences, with P values indicating statistical significance (P < 0.05).

### Attitudes toward medical school

No significant differences were observed between FG and NFG participants regarding their willingness to attend medical school again if revisiting career choices or their attitudes toward medical school.

### Career path after medical school

Career considerations, including factors such as working for social change, high-income potential, social recognition or status, stability, secure future, creativity and initiative, expression of personal values, availability of jobs, leadership potential, work/life balance, ability to pay off debt, and opportunity for innovation after medical schools, showed no significant differences between FG and NFG groups.

### Quality of life and perceived stress

There were no significant differences in QOL and PSS-4 scores between FG and NFG participants (*P* > 0.05).

### Perceptions of medical school experience

Regarding undergraduates’ perceptions toward their medical school, 46.2% agreed that they closely shared most of their classmates' professional values and interests. Approximately one-third (31.4%) agreed that their medical school experience contributed to their ability to work in disadvantaged communities. Additionally, 40% of the students agreed that they often felt that their performance was being judged as a member of the identity group to which they belonged. There was a non-significant difference between FG and NFG undergraduates regarding their attitude toward medical.

### Daily activities and time allocation

An interesting pattern emerged when comparing the daily hours spent on various activities for both groups. Notably, 40.9% of undergraduates allocated 6-8 hours daily to sleep, while 32.1% dedicated 4-6 hours to educational activities. Additionally, 45.4% spent less than four hours on non-educational activities, 76.3% invested less than four hours in paid work, 77.2% in exercise/sports, and 68.1% in other activities. However, a non-significant difference was observed between FG and NFG undergraduates regarding the daily hours spent on these activities.

## DISCUSSION

This study examined the differences between FG and NFG medical students in various aspects related to their educational journey, career aspirations, attitudes toward medical school, and perceived stress levels. FG students were less determined about their future specialty and more inclined toward the USMLE and PLAB exams, essential for ensuring physicians' qualifications in their respective countries. The USMLE comprises three parts—Step 1, Step 2, and Step 3—that medical students and graduates must complete before they can begin and finish their postgraduate clinical residency training in the US [13]. In contrast, international medical graduates (IMGs) face challenges when moving to the UK for training or employment. To practice medicine there, they must pass the PLAB exam, which includes a written (PLAB 1) and practical (PLAB 2) component administered by the General Medical Council (GMC) [14]. FG students desire to pursue the USMLE and PLAB as a means of self-proving, feeling a strong obligation to demonstrate their capabilities due to cultural expectations and family pressure. These exams are important milestones in validating their skills and enhancing their professional opportunities. Also, they exhibited less interest in future teaching roles than NFG students. No significant differences were found in attitudes toward medical school, career considerations, quality of life, or daily activities between the two groups. The study highlighted distinctions in career aspirations and educational preferences between FG and NFG medical students.

When comparing our study results with those of other studies, we found that, as outlined in our research, there is a need to establish robust support programs and create an inclusive environment for trainees, physicians, and patients [1,15]. Prior studies that analyzed sociodemographic factors and the need for creating support programs for FG students prioritized the importance of understanding and accommodating diverse student backgrounds [1,16]. Moreover, some universities have started to provide support systems for FG students. For example, according to a study conducted in 2020, the David Geffen School of Medicine established FG programming to connect FG medical students, residents, and fellows with dedicated FG faculty members for mentorship. The program also provided medical trainees with strategies for self-preservation and well-being. They also provided academic support [2,17].

The present findings were consistent with those of other research that discussed variations in career preferences among medical students. When examining future specialty preferences, notable differences emerged between FG and NFG participants, with a higher percentage of FG students remaining undecided. Further exploration of postgraduate studies and career preferences revealed distinct choices, including a higher preference for exams such as USMLE and PLAB over Saudi Medical Licensing Examination (SMLE) among FG participants. Moreover, FG students showed less interest in future teaching roles [2]. These findings underscored the need to recognize and address diverse career aspirations, particularly among FG medical students. Experiential assets among medical students, particularly FG individuals, played a crucial role in patient care while enhancing peer dynamics and overall quality of care, as well as valuable contributions aligning with the goal of a diverse and patient-centered medical education landscape [2].

Our findings uncovered a range of challenges FG medical students faced, which were also addressed in other studies, from pre-med preparation to navigating the hidden curriculum and social inequalities within medical education. The socio-demographic analysis showed variations in pre-med preparation, mentorship opportunities, and challenges extending into the professional environment, particularly with the shift to pass/fail grading and classism perpetuated by lack of time. Collectively, these factors underscored the complex landscape FG students navigated during their career journey. A previous study on the Canadian pre-med pipeline further emphasized these disparities and suggested solutions, mainly through enhanced mentorship programs [18-20].

Furthermore, various aspects of time management dynamics among medical students were explored, revealing an interesting pattern in the daily hours spent on multiple activities, indicating varied time management approaches. The concept of time as a social asset was discussed in the context of classification, emphasizing the need to understand and address sociodemographic differences in time allocation for a more equitable educational experience [19,20].

The results advocated the urgent need for systemic interventions and policy updates to enhance inclusivity in the medical field [19,20]. Our research emphasizes the importance of promoting an equitable and supportive environment for all medical students and fostering transparent and centralized resources to address the challenges faced by FG students [19].

Acknowledging the limitations of our study, including its cross-sectional design, which examined a specific timeframe without delving into potential personal adaptations and longitudinal changes, the limited sample size, and reliance on self-reported data may have introduced bias. Another limitation of our study was the inability to distinguish between pre-clinical and clinical years for separate analyses. Different educational backgrounds, experiences, and exposure to clinical settings may have affected how coping mechanisms developed for students in these phases. Additionally, the demands and stressors of the clinical years often required the development of new coping strategies that pre-clinical students had not yet developed. Future research could benefit from a more detailed examination of coping mechanisms by considering these distinctions.

Furthermore, future research should broaden its scope to include a more diverse population, encompassing FG and NFG medical students at pre-clinical, clinical, and residential levels. Despite randomly distributing the questionnaire, we encountered challenges obtaining a larger sample size to generalize the results to the targeted population. This approach would uncover comprehensive patterns and differences in their experiences, facilitating the development of tailored support systems and interventions for FG students. These efforts are essential for fostering inclusivity, enriching educational experiences, and enhancing the well-being of students.

## CONCLUSION

This study highlighted the challenges FG medical students face in Saudi Arabia, emphasizing the need for tailored support programs focused on career preferences, financial obstacles, and well-being. This research underscored the importance of inclusivity and targeted interventions to enhance the educational experience of FG medical students. While shared experiences exist across student demographics, recognizing and addressing the unique needs of FG students is crucial for fostering a more supportive and equitable medical education environment.

## Data Availability

Further data are available from the corresponding author upon reasonable request.
